# Supplementary material for: Clinical and Cytogenetic Characterization of Early and Late Relapses in Patients Allografted for Myeloid Neoplasms with a Myelodysplastic Component
Source: Cancers (Basel). 2022 Dec 18;14(24):6244. doi: 10.3390/cancers14246244 (PMC9776604; doi:10.3390/cancers14246244)
Supplement: Supplementary file 1 [file cancers-14-06244-s001.zip › cancers-2105698-Supplementary.pdf]

Supplemental Material: Clinical and Cytogenetic Characterization of Early and Late Relapses in Patients allografted for Myeloid Neoplasms with a Myelodysplastic Component

Victoria Platte, Anika Bergmann, Barbara Hildebrandt, Dagmar Wieczorek, Esther Schuler, Ulrich Germing, Jennifer Kaivers, Rainer Haas, Guido Kobbe, Thomas Schroeder and Christina Rautenberg

Table S1. Relapse Characteristics.

	No.	%
**No.**	91	100
**Time to relapse, median (range), months**	5.5 (0.5 - 110)	
**WBC, median (range), ×10⁹/L**	3.5 (0.4 – 20.2)	
**Type of relapse**		
Molecular	23	25
Hematologic	68	75
**PB Blasts, median (range), %**	0 (0 - 62)	
**BM Blasts, median (range), %**	7 (0 - 90)	
**Median Hb, median (range), g/dL**	11,2 (7 - 17)	
**Platelets, median (range), ×10⁹/L**	87 (0.04 - 800)	
**PB chimerism, median (range), %**	84.2 (0 - 100)	
**BM chimerism, median (range), %**	86 (1,2 - 100)	

BM, bone marrow; Hb, hemoglobin; PB, peripheral blood; WBC, white blood cells.

Table S2. Pretransplant and post-transplant morphology and cytogenetics in 91 patients with myeloid neoplasms.

UPN	WHO at diagnosis	Cytogenetics at diagnosis	WHO at relapse	Cytogenetics at relapse
**1**	MDS-EB I	47,XY,+8[28], der7, del7q	MDS-EB I	47,XY,+8[3]//46,XY[1]/46,XX[20]
**2**	AML-MRC	47,XX,+8[17]/46,XX[5]	AML-MRC	47,XX +8[18]/47, idem, t(3;7)(q;q),add(17q)[2]/46,XY[2]/46,XX[1]
**3**	MDS-EB II	45,XX,t(3,3)(q21;q26),-7[17]/46,XX[3]	AML	missing
**4**	AML-MRC	47,XY,+8	AML	46,XY [24]
**5**	AML-MRC	46,XX,add(5)(q31),der(5)t(5;12)(q13;q13),-6,-12,der(20)(q11q13),add(21)(q22),-21,+mar1,+mar2,+mar3[14]	AML-MRC	46, XX, add(5)(q12), add(5)(q33) del(6)(q13) del(20)(q11)[3]/ 46; XY[18]
**6**	MDS-MLD	46,XY	MDS-EB II	46, XY
**7**	MDS-MLD	45,XY,del(3)(q1?),+4,der(5;17)(p11;q11),-7,+8,-18,-22[13]/46,XY[20]	MDS-MLD	46,XY[22]
**8**	CMML II	46,XY[12]/del(10p)	CMML 0	46,XX[14]/46,XY[10]
**9**	MDS-EB II	46,XY	AML	46, XY
**10**	CMML I	47,XX +8	AML-MRC	47,XX+8 [3]

**11**	MDS-EB II	46,XX,t(3;3)(q21;q26), del(7)(q11;1) [15], 46 XX, [5]	MDS-EB I	46,XY[25], FISH: -7q (9,5%); -7 (10,5%)
**12**	MDS-EB II	46,XX,del(9)(p22)[9]/46,XX[14]	MDS-MLD	del (9)(p22)
**13**	CMML I	46,XY [20]	CR, molecular relapse	46,XY[4]/46,XX[19]
**14**	MDS-EB I	46,XY	MDS-EB I	46,XY[20]/del(7q)//-Y[2]
**15**	MDS-EB II	45,XY,der(5)t(5;20)(q11;?),+der(5)t(5;20)(q11;?), -13,-18,-20,+mar[1]/46,XY[22]	CR, molecular relapse	43,XY,der(5;20),-13,-18[1]/45,XY,+Y,der(5;20)x2,-13,-18[1]/46,XY[23]
**16**	MDS-EB II	t(2;11)(p21;q23);del(5q)(q22q33)[20]/46, XY [3]	MDS-EB I	47,XY,t(2;11)(p21;7q23),del(5)(q22q33),add(19)(q13),+21[12]/46,XY811]
**17**	MDS-EB II	46,XX	MDS-EB II	46,XX[5]/46,XY[14]
**18**	MDS-EB I	47,XY,+6[4]/46,XY[21]	MDS-EB I	46XX[7]/48,XY,+8,+21
**19**	MDS-EB II	45,XY,del(5)(q22q34),-7,t(6;21)(p25;q11)[2]	MDS-EB I	45,XY,del(5)(q22q33),-7,+19,der(20;22),-22,+2mar[9]/44,Xder,der(12;14)(q11;q11)[1]/44,idem,t(1;2)(p13;q35),der(12;14)(q11;q11)[9]/46,XY[4]
**20**	AML-MRC	47,XX,+8 (17)/46, XX (5)	AML-MRC	47, X, X, t(1,12) (p21;q24), t(3;5) (q12;p15) +8, der (12) [20] / 46, XX [4]
**21**	AML-MRC	53,XY,+4,t(6;11;14)(q27, q23,q24),+9,+13,+16,+21,+22	AML	missing
**22**	MDS-EB II	46,XY	AML	missing
**23**	MDS-EB I	46,XY,+1,+der(1;14)(q11;q11)[20]/46 XY[2]	MDS-MLD	46,XY, +1,der(1;14)(q10;p10)[19]/46,idem,del (20)(q11)[2][46,XY[2]
**24**	AML-MRC	XY,add(8)(q24),del(11)(q13),del(17)(p11),+19,+19,+20,+21 [11]/46, XY[13]	AML-MRC	XY, add(8)(q24), del(11)(q13), del(17)(p11), +19, +19, +20, +21 [11]/ 46, XY [13]
**25**	MDS-EB II	44-46,XY,t(2;?3)(p23~25;q12~21),-5,add12(p1?),-17,-19,+1~3mar[19]/88~90,idem x2[2]/46,XY[4]	CR, molecular relapse	FISH: del17p, del5q
**26**	AML-MRC	46,XX	CR, molecular relapse	46, XY
**27**	MDS-EB I	44-46,XY,-5,der(6),t(6;17)(p23;q11),+1-2mar[24] 46,XY [8]	MDS-MLD	44, XY, -5, der(6)t(6;17)(p25;q11),mar[2]/ 46, XX[26]
**28**	AML-MRC	51,XX,71,del(5)(q22;q33),+11,+14,+15,-21	AML-MRC	46,XY[21]
**29**	MDS-MLD	46,XX,del(5)(q22q33),del(6)(q23),del(20)(q13)[22]/46,XX[2]	MDS-MLD	46,XX,del(5)(q22q33),del(6)(q23),del(20)(q13)[22]/46, XX[2]
**30**	AML-MRC	45,XY,-7[20]	MDS-EB I	45,XY,-7[2],26,XY[24], FISH 5,2%
**31**	AML-MRC	43,XY,-7,-12,-14,del(5q),del(16)(q13)	MDS-EB I	43,XY,del(5)(q22q34),-7,-12,-14,del(16)(q13),add(18)(q23) [5]/44,idem,+(3;?)(p12;?) [3]/46, XY[17]
**32**	MDS-MLD	46,XX	AML	46, XX, t(9;22)(q34;q11) [28]
**33**	AML-MRC	47,XY,+8	CR, molecular relapse	47,XY,+8
**34**	MDS-EB I	46,XX	molecular relapse	46,XY
**35**	AML-MRC	46,XY,t(1;12)(q21;p12)[21]/48,idem,+8,+8[4]	CR, molecular relapse	46,XY,t(1;12)(q21;p12)[21]/48,idem,+8,+8[4]
**36**	AML-MRC	42,XX,der(9,17)(p10;q10),der(11)dyp(11)(q13q23),t(11,13)(q23,q11) der(16),-18[18]/46,XX [6]	CR, molecular relapse	46,XY[23]

**37**	AML-MRC	49~51,XY,+Y,+12,+21,+del(12)(q21q22), +idic(21)(q22)x1~3[cp9]	AML-MRC	51,XY,+12,+21,+21,+21 [12]
**38**	CMML II	46,XY	AML-MRC	46,XY,add(1)(q32)[10]/46,XY,t(18;21)(q12.1.q13)[9]/46,XY[4]
**39**	AML-MRC	46,XX,add(1)(p13), der(1;8)(p10;p10),der(5)t(5;8)(q13;q22), -20,20~25dmin[20],46,XX[1]	AML-MRC	46; XX, del (1) (p22), der(5) t(5;?) (q22;?), + der(8) t(1;8) (q22;q22), add(12) (p11) ? del (16) (q23) add (17) (q11), -20, -22[12], + mar [28]
**40**	MDS-EB II	46,XY[23]	CR, molecular relapse	44, XY, del(4) (q31), -5, del(7) (q22), add (9) (p21), add (17) (q11), add (20) (p12),-21, -22, +r[5], + mar[6][22]/46 XY[4]
**41**	MDS-EB II	46, XY	CR, molecular relapse	46,XY[25]
**42**	MDS-EB II	46, XY	MDS-EB I	46, XY, del(3)(q21q25)[18]/47idem+8[5
**43**	MDS-EB II	47,XX,der(3)t(3;?4)(p21;q23)add(3)(q29),del(4)(q21q?33),del(5)(q21q34),+mar1[10]/46,al,-17,-mar1,+mar2[5]/45,adl1,-21,-mar2,+mar3[2],FISH: 48,5% Inversion 3; 78,6% Deletion 5q; 26,8% Deletion 17p13 und 17p11	MDS-EB I	46,XY[10]/47,XX,der(3)t(4;3;5),del(4)(q21),del(5)(q22;q33),+7,-17,+mar[3]
**44**	AML-MRC	40-42, XY,del(2)(q?31),der(4)t(4;13)(q27;q?)del(5)(q11),-7,-13,add(17)(q23),-19,-20[2]/46,XY[27]	AML-MRC	42, XY, del(2) (q31), der(4) t(4;13) (q? 27;q?11), del(5) (q11q33), -7, -13, add(17) (q23) -19, -20 [21]
**45**	AML-MRC	46, XY	AML-MRC	46,XY[24]
**46**	MDS-EB I	46,XY,del(5)(q22q23),-7,del(12)(p11),+mar[21]/46,XY[3]	MDS-MLD	46,XY,del(5)(q22q23),-7,del(12)(p11),-18,add(19)(q13),+mar[13]/46,idem,del(17)(p11)[3]
**47**	MDS-u	46,XX,t(1;5)(p31;q?33)[20]/46,XX	MDS-EB II	46,XX
**48**	CMML I	47,XX,+8,del(12)(p12)[11]/48,idem,+add(9)(q11)[5]/50,idem,+9,+13,+22[8]	CMML II	47,XX,del(6)(q21),+8,del(12)(q21),add(20)(p11)[15]/46,XY[9]
**49**	MDS-MLD	46, XY	MDS-EB II	46,XY
**50**	AML-MRC	46, XY, der(4)t(4;13)(p16;q14), del(12)q(21),-13, -15, del(22)(q11), der(?) t(1;?)(p22;?),+mar [18]	molecular relapse	46, XY, der(4)t(4;13)(p16;q14), del(12)q(21),-13, -15, del(22)(q11), der(?) t(1;?)(p22;?),+mar [18]
**51**	MDS-EB II	46, XX, del(20)(q11)[20], 46, XX[6]	MDS-MLD	46,XX,del(20q)(q11)[3]/46,XY[22]
**52**	MDS-EB II	49, XX, +8, +12, +13, i (17)(q10)(4), 46, XX (12), FISH:	CR, molecular relapse	48, XX, +8, +13, i(17) (q10) [6] 46, XX [18]
**53**	MDS-EB II	42-47, XY, add(1) (p36), der(3), del(3) (p21p11) add(3) (q26), -5, -6, -8[8], add(9) (q34) [4], add(17) (p11), -18, +22[3], +2-4 mar [14] / 46, XY	AML	46,XY[28]
**54**	MDS-EB II	46, XY	CR, molecular relapse	46,XY
**55**	MDS-u	47, XY +8[5], 46 XY[19] und zudem 45 XY-7[2]	MDS-SLD	46, XY
**56**	CMML II	46, XY	MDS-EB II	FISH: Del 21p
**57**	MDS-EB II	46,XY	MDS-EB I	46,XY, t(2;21)(q31;q22),der(9q)[20], 45,X, -Y [1], 46, XY[3
**58**	MDS-EB II	46, XX, +1, del(5)(q14q34),der(8)t(8;12)(q22;q22),der(12)t(8;12)(q24;p12)t(8;12)(q22;q22),der(13;17)(q10;q10), FISH: Deletion 5q31, Deletion ETV6, Deletion TP53/17p	CR, molecular relapse	42,X,-X,+del(1)(q21),del5)(q22q33),-7,del(8)(q?),add(11)(q23),del(12)(p13),der(13;17)(q10;q10),-16,-17,der(21),-22[4]/46,XX[20]

**59**	MDS-EB I	46,XY,+1, del(6)t(1;6)(q21;p23)[16]/46,XY[14]	CR, molecular relapse	t(12;21)(p11;p11)
**60**	MDS-RS	46, XX	MDS-EB II	46,XX
**61**	CMML 0	47, XY +21	molecular relapse	47,XY,+21[13]/46,XX[11], FISH 39,2
**62**	MDS-EB II	47, XX, +8, +9	MDS-EB I	46,XX[12]//46XY[20]
**63**	AML-MRC	46,XX,del(7)(q22q23)[2]/46,XX[23]	CR, molecular relapse	46,XX,del(7)(q22)[8]/46,idem,add(15)(q24)[5]/46,XX[11]
**64**	AML-MRC	46,XY	AML	46, XY [1] / 46, XX [23]
**65**	MDS-EB I	46,XX, t(2;3)(p23;q26),del(7)(q22q36)[12], 46, XX [2]	CR, molecular relapse	46,XX, t(2;3)(p23;q26),del(7)(q22q36)[17], 46, XX [3]
**66**	MDS-EB II	46, XX	AML	45, XX, der(6;17) (p11,q11), -6 [19]// 46, XX [4]
**67**	MDS-EB II	45,X-Y,inv(3)(q21q26)[1],46,XY[19]	CR, molecular relapse	45,X,-Y,inv(3)(q21q26),t(4;12)(q12;p12)[21]/ 46,idem,+8[2]
**68**	MDS-EB II	46,XY,del(5)(q1?3q3?1)[4]/45,XY, del(5)(q1?3q3?1),-7[12]/46,XY[8]/ 45,XY, del(5)(q1?3q3?1),-7,+21[1]	MDS-MLD	46, XY, del (5) (q22q33) -7, +21 [2]<br>46, XY [22]
**69**	AML-MRC	45,XY,-1,del(5)(q11),add(9)(p11),-9, del(11)(q21),del(12)(p11),-15,-17,+mar1,+mar2,[13]/45,XY,del(1)(q32), del(5)(q11),add(9)(p11),-12,-13,add(14)(p11),-15,-17,+mar1,+mar3[11]/ 46,XY[3]	AML-MRC	43-44, XY, del(1)(q32), del (5)(q11), add (9)(p24), del(9)(q22), add(11)(p15), add(12)(p11), add(12)(q24), -15, -17[9] / 46, XY [23]
**70**	MDS-EB I	46, XY	CR, molecular relapse	46,XY
**71**	AML-MRC	46,XX,del(7)(q32)	AML-MRC	46, XX, del(7)(q32), del(11) (p15) [4] / 46, XY [20]
**72**	CMML I	46, XY	MDS-MLD	46, XY, t(1;2) (p36,p21) [22]<br>46, XY [2]
**73**	AML-MRC	46,XY[24]	AML-MRC	(73,4%) 46, XY [22] \ \ 46, XX [8]
**74**	MDS-MLD	47, XY +8	AML	47, XY, +8, +(8;16)(p11;p13) [9] /<br>46, XY [6] / 46, XX [9]
**75**	AML-MRC bis undifferenziert	46,XX[26]	MDS-EB II	46,XX[24]
**76**	MDS-EB I	45,XY,-7[19]	MDS-EB I	46,XY[26]
**77**	AML-MRC	46,XY	CR, molecular relapse	46, XY [24]
**78**	MDS-EB II	46, XY	AML	46,XY
**79**	MDS-EB I	46, XY	MDS-EB I	46, XY [20]
**80**	MDS-EB I	46, XY	molecular relapse	46, XX
**81**	MDS-EB I	46, XY	AML-MRC	46,XY[12]/46,XX[12]
**82**	AML-MRC	43-46 XX, t(3;11), del(5q), -7, add(14)(q11), der(18), -19, -21, +2mar[20]/46,XX[4]	MDS-EB I	43-46 XX, t(3;11), del(5q), -7, add(14)(q11), der(18), -19, -21, +2mar[20]/46,XX[4]
**83**	AML-MRC	46, XY	AML	46,XY
**84**	MDS-MLD	47,XX + 8,del(5q)(14q31)	MDS-EB I	del(5q)(14q31)

**85**	MDS-MLD	45,X, -Y	MDS-MLD	46, XY
**86**	AML-MRC	45,XX,-7	MDS MLD	45, XX, -7
**87**	AML-MRC	47,XX,+21[7]/ 46,XX[14]	MDS-EB II	46,XX,add(5)(q35),del(7)(p15)[9]/46,XY[15]
**88**	MDS-EB II	46,XX,t(8;12)(q24;q13),del(20)(q11q13),add(17)(p13)	MDS-EB II	46, XX, t(8;12) (q24;q11), add(17) (p11), der(20) / [19] / 46, XX [5]
**89**	MDS-EB II	48, XXY, +8, add(21p)	CR, molecular relapse	48, XXY, +8, add(21p)
**90**	MDS-u	48, XX, +4, +11 [11]	MDS-MLD	46, XX[4]/46, XY[27]
**91**	MDS-EB I	46, XY	CR with MLD/MDS-EB I	missing

AML, acute myeloid leukemia; AML-MRC, acute myeloid leukemia with myelodysplasia related changes; CMML-0/-I/-II, chronic myelomonocytic leukemia; CR, complete remission; MDS-EBI-/EBII, myelodysplastic syndrome with excess of blasts I/II; MDS-MLD, myelodysplastic syndrome with multilineage dysplasia; MDS-SLD, myelodysplastic syndrome with single lineage dysplasia; MDS-RS-MLD, myelodysplastic syndrome with multilineage dysplasia and ring sideroblasts; MDS-RS-SLD, myelodysplastic syndrome with single lineage dysplasia and ring sideroblasts; MDS-u, myelodysplastic syndrome unclassifiable; WHO, world health organization.

Table S3. Treatment of Relapse and Response.

		Response	No Response
**Early Relapse**	**n=56**		
Intensive salvage	15	4	11
Non-intensive salvage	41	16	25
**Late Relapse**	**n=35**		
Intensive salvage	10	5	5
Non-intensive salvage	25	17	8

Intensive salvage therapy represents chemotherapy-based salvage regimens such as FLAG-Ida or high dose cytarabine and mitoxantrone and/or second allogeneic stem cell transplantation. Non-intensive salvage therapy represents chemotherapy-based salvage regimens such as Hypomethylating agents +/- Venetoclax +/- Donor Lymphocyte Infusions.
